# Metabolic risk factors and the incidence and progression of radiographic hand osteoarthritis: a population-based cohort study

**DOI:** 10.1080/03009742.2018.1459831

**Published:** 2018-06-28

**Authors:** M Marshall, G Peat, E Nicholls, HL Myers, MA Mamas, DA van der Windt

**Affiliations:** 1 Arthritis Research UK Primary Care Centre, Research Institute for Primary Care and Health Sciences, Keele University, Staffordshire, UK; 2 Arthritis Research UK Primary Care Centre, Research Institute for Primary Care and Health Sciences and Keele Clinical Trials Unit, Keele University, Staffordshire, UK; 3 Keele Cardiovascular Research Group, Guy Hilton Research Centre, Keele University, Stoke-on-Trent, UK

## Abstract

**Objective**: To determine whether selected metabolic factors are associated with greater amounts of radiographic hand osteoarthritis (OA) incidence and progression.

**Methods**: The study identified 706 adults, aged 50–69 years, with hand pain and hand radiographs at baseline, from two population-based cohorts. Metabolic factors (body mass index, hypertension, dyslipidaemia, and diabetes) were ascertained at baseline by direct measurement and medical records. Analyses were undertaken following multiple imputation of missing data, and in complete cases (sensitivity analyses). Multivariable regression models estimated associations between metabolic factors and two measures of radiographic change at 7 years for all participants, individuals free of baseline radiographic OA, and in baseline hand OA subsets. Estimates were adjusted for baseline values and other covariates.

**Results**: The most consistent and strong associations observed were between the presence of diabetes and the amount of radiographic progression in individuals with nodal OA [adjusted mean differences in Kellgren–Lawrence summed score of 4.50 (−0.26, 9.25)], generalized OA [3.27 (−2.89, 9.42)], and erosive OA [3.05 (−13.56, 19.67)]. The remaining associations were generally weak or inconsistent, although numbers were limited for analyses of incident radiographic OA and erosive OA in particular.

**Conclusion**: Overall metabolic risk factors were not independently or collectively associated with greater amounts of radiographic hand OA incidence or progression over 7 years, but diabetes was associated with radiographic progression in nodal, and possibly generalized and erosive OA. Diabetes has previously been associated with prevalent but not incident hand OA. Further investigation in hand OA subsets using objective measures accounting for disease duration and control is warranted.

Symptomatic hand osteoarthritis (OA) is estimated to affect 8.2% of men and 15.9% of women in the general population (1). The course of hand OA is not clear but is thought to be heterogeneous, with some individuals experiencing substantial deterioration in structure, pain, and function while others remain stable for many years (2). Currently, there is limited evidence regarding the risk factors for hand OA progression (3), and the need to gain further understanding of the aetiology and course of hand OA has been highlighted as a research priority (4).

Metabolic factors have been associated with hand OA, but mainly in cross-sectional studies; systematic reviews have reported associations of obesity and type 2 diabetes with hand OA (5, 6), and additional studies have reported associations between metabolic syndrome and hand OA (7–9). These findings suggest that systemic metabolic disturbances may play a role in the pathophysiology of hand OA. As the hands are not exposed to the joint-loading effects of obesity, they are an ideal site to investigate associations between metabolic factors and OA.

However, the role that metabolic factors play in the incidence and progression of hand OA is unclear, as little longitudinal research has been undertaken. Apart from one study that did not find an association between type 2 diabetes and incident hand OA (10), and two studies that examined hyperlipidaemia and incident hand OA with differing results (11, 12), most studies have focused on obesity, and conflicting findings have been reported (13–22). The disparity in results could be due to variations in study populations and the definitions of progression used. However, the relationship between obesity and hand OA progression could be confounded or mediated by the presence of hypertension, dyslipidaemia, and diabetes; and to date, few analyses have examined metabolic factors independently from each other. Furthermore, the impact of multiple metabolic factors on the course of hand OA has been examined, but only in a single study where no association was found between metabolic syndrome and incident and progressive hand OA (23).

There is some evidence that the role of metabolic factors in hand OA pathogenesis varies between subsets of hand OA. Obesity, hypertension, dyslipidaemia, and metabolic syndrome occurred more often in community-dwelling individuals with erosive OA than in other subsets (24). Also, significantly elevated levels of a serum adipokine adiponectin, which have been associated with obesity, have been found in erosive OA compared to patients with non-erosive hand OA and healthy controls (25). The association between atherosclerosis and hand OA progression was noted to differ by joint group (26). Therefore, the conflicting findings previously reported between obesity and hand OA incidence and progression could also be explained by differing proportions of hand OA subsets within the study populations.

This study sought to determine, in population-based older adults, whether obesity, diabetes, hypertension, dyslipidaemia, and the accumulation of metabolic factors are independently associated with radiographic hand OA incidence and progression over 7 years, as well as progression within different baseline subsets of hand OA.

## Method

### Study population and design

Study participants were from a population-based prospective cohort, the Clinical Assessment Studies of the Hand (CASHA) (27). At baseline, all adults aged ≥ 50 years registered with two general practices in North Staffordshire, UK, were invited to participate in a two-stage survey. In the UK, 95% of people are registered with general practices, thus providing convenient general population sampling frames. Those reporting hand pain or problems in the past year were invited to attend research clinics that included radiographs and assessment of finger nodes in the second and third interphalangeal joints (IPJs) of each hand by trained assessors. To increase the numbers in each hand OA subset, the sample was enriched with participants from the Clinical Assessment Studies of the Knee (CASK) (28) who were recruited using an identically performed two-stage survey in a similar population of three general practices in the same locality of North Staffordshire. Individuals in this study who reported knee pain in the previous year were invited to attend research clinics, where they also received identical hand radiographs and assessment of finger nodes to CASHA participants.

All participants included in the current analyses were aged 50–69 years at baseline, had reported hand pain on a few days or more in the previous month (29), had hand radiographs, and did not have inflammatory arthritis (n = 764). Follow-up was at approximately 7 years with a postal questionnaire and research clinic including hand radiographs. An Index of Multiple Deprivation (IMD) (30), based on a combination of education, employment, income, health, and crime figures in English neighbourhoods, was obtained for each participant using their postcode. UK Local Research Ethics Committees approved these studies (LREC project numbers: 1430, 05/Q2604/72, 06/Q2801/90). All participants provided written informed consent.

### Radiographic assessment

Posterior–anterior hand radiographs were taken according to a standardized protocol (27, 28). Two trained readers scored 20 hand joints [distal, proximal, and thumb IPJs, and first carpometacarpal joint (1CMCJ)] for OA using the Kellgren–Lawrence (KL) grading system (0–4) at baseline and 7 years, unpaired but with known chronological order (31). A single reader scored the presence of erosive OA using the Verbruggen–Veys Anatomical Phase Progression Score in 16 IPJs at each time-point (32). Reliability has previously been reported for the presence of OA and erosive OA at baseline (24). At 7 years, intra-rater reliability was substantial for OA [unweighted kappa (K_u_) = 0.88 and 0.67, percentage agreement (PA) = 96% and 93%] and erosive OA (K_u_ = 0.89, PA = 99%), and inter-rater reliability was moderate for OA (K_u_ = 0.64, PA = 91%) and substantial for erosive OA (K_u_ = 0.84, PA = 98%).

### Hand OA subsets

The hand OA subsets were examined and their definitions were: thumb base OA, KL ≥ 2 in the 1CMCJ in either hand; nodal IPJ OA, KL ≥ 2 in ≥ 2 IPJs (rays 2–5) and ≥ 2 nodes (rays 2–3) across either hand; generalized hand OA, KL ≥ 2 in ≥ 1 distal IPJ and ≥ 1 proximal IPJ and ≥ 1 1CMCJ across either hand; and erosive OA, E- or R-phase of the Verbruggen–Veys Anatomical Phase Progression Score in ≥ 2 IPJs (rays 2–5) across either hand (24).

### Radiographic change

The amount of radiographic change was assessed by two outcomes, using continuous measures to avoid loss of information and inflation of type 2 errors (13, 25, 33, 34):

- the KL summed (KLsum) score for 20 hand joints at 7 years (0–80) adjusted for the baseline KLsum score (0–80) (providing a composite measure of change that combines the amount of within-joint change and the number of joints changing)
- the number of joints with KL ≥ 2 at 7 years (0–20) adjusted for baseline number of joints with KL ≥ 2 (0–20) (change represents the number of joints newly classed as having definite radiographic disease).

Incident radiographic OA was investigated in participants free of radiographic OA (KL < 2) at baseline, whereas the term progression was used to collectively refer to radiographic worsening in participants with and without baseline radiographic OA.

Participants with maximum scores at baseline were excluded from the analyses, as they could not undergo further progression.

### Risk factors

Metabolic risk factors included body mass index (BMI), determined from height and weight measured at the baseline research clinics and used as a continuous variable. Consultations and/or diagnoses of hypertension, dyslipidaemia, and type 2 diabetes/impaired fasting glucose (IFG) in the 2 years before and 2 years after the baseline research clinics were obtained from primary care medical records for those participants providing permission (94%, n = 660) and used as dichotomous variables. Consultations and diagnoses in the UK are coded using a hierarchical method of standardized Read Codes (35). The validity of using the Read Codes for type 2 diabetes and hypertension was examined against individuals self-reporting having diabetes and raised blood pressure, and was found to be 95% and 87%, respectively. The validity of having a Read Code for type 2 diabetes was further checked against prescription records. All individuals prescribed a diabetic drug in the 2 years before and after baseline had a Read Code for diabetes in the same period.

The collective influence of multiple metabolic factors was examined using the number of metabolic risk factors (0–4) and the presence of metabolic syndrome (adapted from the NCEP/ATPIII definition) (36), which was classed as three or more of the following: BMI ≥ 30 kg/m^2^, hypertension, dyslipidaemia, and diabetes type 2/IFG.

### Statistical analysis

Descriptive characteristics for all baseline participants, and those followed up at 7 years by postal questionnaire and at the 7 year research clinics were compared.

Baseline scores were plotted against 7 year scores to investigate the amount of radiographic change for the two outcomes, stratified by gender. Adjusted mean estimates and 95% confidence intervals (CIs) were determined at 7 years for both outcomes using analysis of covariance. Analyses were stratified by gender and adjusted for baseline value of the outcome, cohort (CASK or CASHA), age, and time to follow-up.

The independent associations between metabolic factors and incidence and progression of radiographic hand OA at 7 years were estimated using analysis of covariance (KLsum, number of joints) using three models: (i) BMI, hypertension, dyslipidaemia, and diabetes type 2/IFG; (ii) metabolic syndrome; and (iii) number of metabolic factors. All models were adjusted for the following potential confounders: baseline radiographic score, gender, baseline age, cohort, time to follow-up, baseline smoking status (never, ex, current), and IMD. This analysis was undertaken in all study participants, stratified by baseline hand OA subset, and in those with no baseline hand OA (KL ≤ 2).

Analyses that exclude individuals with missing data are acknowledged to produce biased estimates and reduced power and precision compared to those including all individuals (37, 38). Multiple Imputation (MI) is recognized as an appropriate statistical method for handling missing data and overcoming the aforementioned limitations through addressing the uncertainty around missing values by generating imputes in multiple data sets (39). Therefore, MI was undertaken using chained equations in all eligible individuals. Primary analyses were undertaken in the imputed data sets, with complete case analyses undertaken for sensitivity purposes (40). Data were imputed for missing 7 year outcome scores (45.0%) and for missing baseline data (BMI 0.3%, IMD 0.3%, baseline smoking status 1.3%, and metabolic factors 6.5% in those not consenting to medical record review). Fifty imputed data sets were generated (41, 42). A relatively large amount of outcome data was imputed, but research has shown that models still perform well in these situations (39, 43–45). The distribution of variables in the imputed data sets were checked, ensuring that plausible values had been imputed, and model assumptions were verified. MI relies on variables being missing completely at random or missing at random (37, 46). Missing data were associated with a number of baseline variables and therefore assumed to be missing at random. The imputation model included these baseline variables as well as the metabolic factors and 7 year outcomes to increase the power and precision of the imputation model (Supplementary Table S1) (47). Data could still be missing as a result of other unaccounted variables, but our participants were well characterized with an extensive range of descriptive, sociodemographic, hand symptom, general, physical and mental health, and self-reported comorbidities. Rubin’s rules were used to combine estimates from imputed data sets (48). Analyses were performed using SPSS version 21.0 (IBM Corp., Armonk, NY, USA).

## Results

### Study population

**Figure 1.**
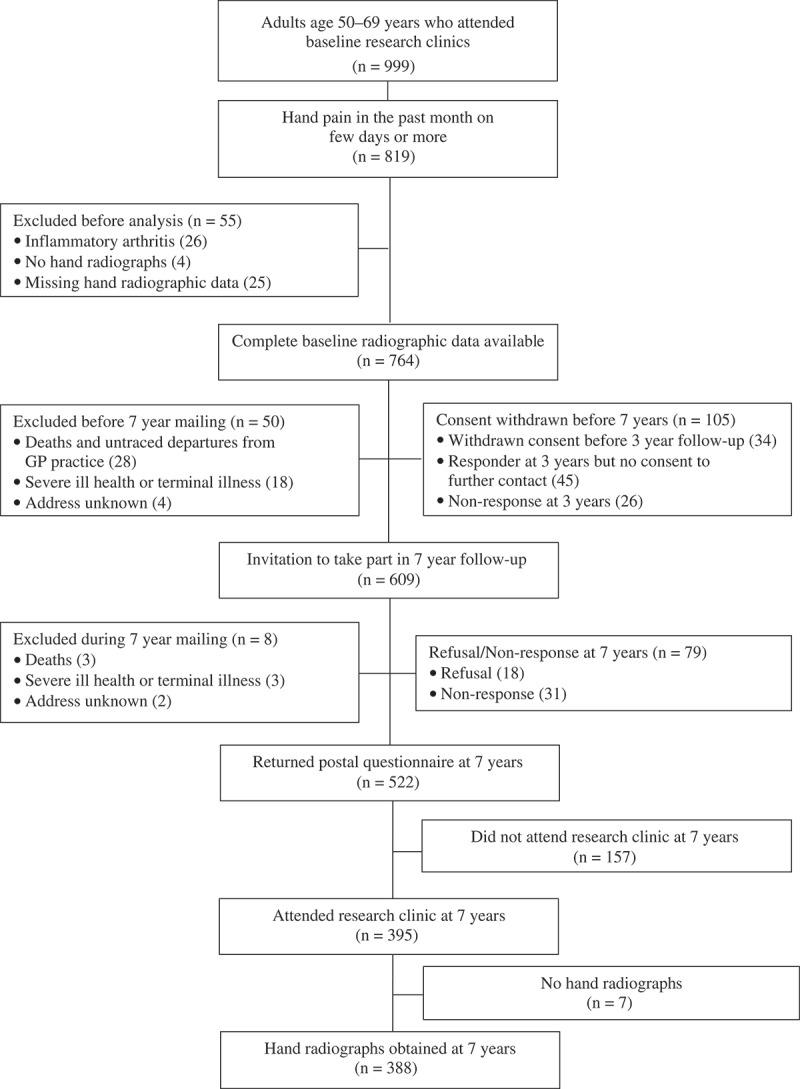
Flow diagram of study participants.

Of 764 eligible individuals at baseline, after 58 exclusions (31 deaths or untraced departures from GP practice, 21 with severe ill health or terminal illness, and six with address unknown), 552 of the 706 were followed up at 7 years (adjusted response 78%) (Figure 1). Those lost to follow-up were mainly due to failure to renew consent to further contact at the interim 3 year follow-up (105). Some respondents at 7 years were unwilling to attend the 7 year research clinic (157); therefore, a total of 388 had hand radiographs at baseline and follow-up, with a mean follow-up time of 83 months (sd 6.7).

Compared with all eligible participants at baseline, those followed up with hand radiographs at 7 years were less likely to be a current or ex-smoker, or to have type 2 diabetes/IFG, and had slightly lower anxiety and depression scores. The distribution of other baseline variables was similar (Table 1).

**Table 1.** Characteristics of study participants overall, those followed up at 7 years, and those with hand radiographs at baseline and 7 years.

Baseline characteristics	Participants (n = 706)	Participants followed up by questionnaire at 7 years (n = 522)	Participants who attended research clinic and had hand radiographs at 7 years (n = 388)
% Female (n)	62.0 (438)	60.9 (318)	60.1 (233)
Age (years), mean ± sd	60.5 ± 5.2	60.3 ± 5.3	60.5 ± 5.2
% CASHA study (n)	51.4 (363)	54.6 (285)	51.5 (200)
Index of Multiple Deprivation, mean ± sd	14 971 ± 7425	15 524 ± 7390	15 316 ± 7509
% White ethnicity (n)	99.7 (693)	99.8 (512)	99.7 (381)
% Smoking (n)			
Never	48.4 (338)	51.8 (268)	54.4 (210)
Ex	41.4 (289)	39.7 (205)	38.6 (149)
Current	10.2 (71)	8.5 (44)	7.0 (27)
% Hand pain on most or all days in the past month (n)	44.7 (315)	44.4 (232)	45.4 (176)
AUSCAN pain, mean ± sd	6.7 ± 4.2	6.5 ± 4.1	6.5 ± 4.1
AUSCAN function, mean ± sd	10.0 ± 8.1	9.6 ± 7.9	9.6 ± 7.9
AUSCAN stiffness, mean ± sd	1.2 ± 1.0	1.2 ± 1.0	1.2 ± 0.9
% Radiographic hand OA (KL ≥ 2 in ≥ 1 joints) (n)	68.7 (485)	68.1 (356)	67.5 (262)
Baseline summed KL score (0–80), mean ± sd	8.2 ± 9.6	7.9 ± 9.2	7.8 ± 9.1
Median (IQR)	5 (2, 11)	5 (2, 11)	5 (2, 11)
Baseline number of joints KL ≥ 2 (0–20), mean ± sd	2.8 ± 3.4	2.7 ± 3.3	2.7 ± 3.3
Median (IQR)	2 (0, 4)	2 (0, 4)	2 (0, 4)
% Thumb base OA (KL ≥ 2 in either 1CMCJ) (n)	42.9 (303)	43.1 (225)	43.6 (169)
% Nodal IPJ OA (KL ≥ 2 in ≥ 2 IPJs (rays 2–5) and ≥ 2 nodes (rays 2–3) across either hand) (n)	21.5 (152)	21.3 (111)	21.9 (85)
% Generalized hand OA (KL ≥ 2 in ≥ 1 distal IPJ and ≥ 1 proximal IPJ and ≥ 1 1CMCJ across either hand) (n)	11.8 (83)	10.9 (57)	11.6 (45)
% Erosive OA (E- or R-phase in ≥ 2 IPJ (rays 2–5) across either hand) (n)	3.1 (22)	3.3 (17)	2.8 (11)
Metabolic factors			
BMI (kg/m^2^), mean ± sd	29.2 ± 5.2	28.9 ± 4.9	28.9 ± 4.9
% Hypertension (n)	36.4 (240)	36.7 (180)	36.6 (234)
% Diabetes type 2 or impaired fasting glucose (n)	10.8 (71)	8.6 (42)	9.8 (36)
% Dyslipidaemia (n)	30.8 (203)	31.4 (154)	31.2 (115)
No. metabolic factors, mean ± sd	1.1 ± 1.0	1.1 ± 1.0	1.1 ± 1.1
Median (IQR)	1 (0, 2)	1 (0, 2)	1 (0, 2)
% Metabolic syndrome (n)	11.2 (74)	10.0 (49)	11.4 (42)
SF-12 Physical Component Score, mean ± sd	39.2 ± 12.1	39.9 ± 11.9	40.0 ± 12.0
SF-12 Mental Component Score, mean ± sd	49.9 ± 11.0	50.8 ± 10.6	51.1 ± 10.6
HADS Anxiety scale, mean ± sd	7.2 ± 4.2	6.8 ± 4.1	6.6 ± 4.1
HADS Depression scale, mean ± sd	4.7 ± 3.6	4.4 ± 3.5	4.3 ± 3.4
SF-36 Physical functioning scale, mean ± sd	60.6 ± 28.4	63.0 ± 27.8	62.6 ± 28.3

CASHA, Clinical Assessment Studies of the Hand; AUSCAN, Australian–Canadian Hand Osteoarthritis Index; OA, osteoarthritis; KL, Kellgren–Lawrence; IQR, interquartile range; 1CMCJ, first carpometacarpal joint; IPJ, interphalangeal joint; BMI, body mass index; SF-12, 12-item Short Form Health Survey; SF-36, 36-item Short Form Health Survey; HADS, Hospital Anxiety and Depression Scale.

One individual was excluded from the analyses examining the progression of the number of hand joints with KL ≥ 2 owing to having the maximum number of 20 joints affected at baseline; where applicable, this is indicated in the relevant results tables.

### Radiographic change

**Figure 2.**
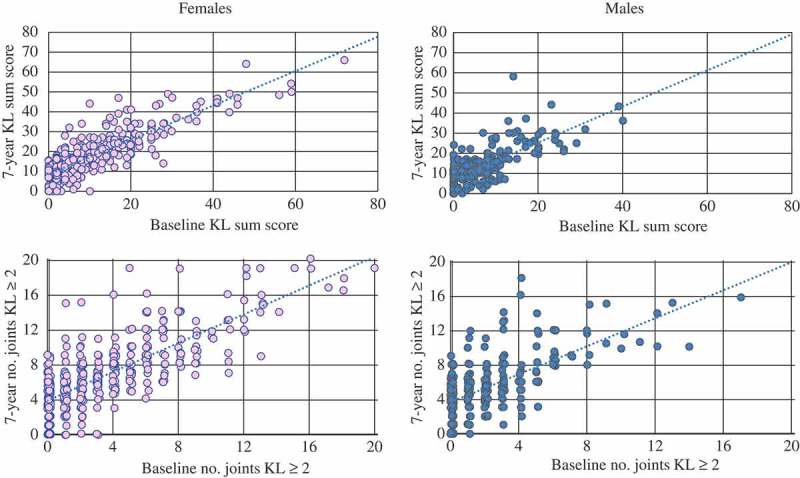
Scatterplots showing the relationship between baseline and 7 year radiographic cores, stratified by gender in the imputed data (n = 706). Jittering has been used to allow better visualization of overlapping markers. KL, Kellgren–Lawrence.

Scatterplots indicate positive linear trends between the baseline and 7 year scores for both hand OA outcomes in the imputed data, and were similar for men and women (Figure 2). Overall, in the imputed data the amount of radiographic change at 7 years was significantly lower in men than in women for each outcome (Table 2). Compared to the overall estimates of radiographic change at 7 years, those who were free of radiographic OA at baseline on average underwent less change, whereas those who had thumb base, nodal, generalized, and erosive OA at baseline experienced more change (Table 2). Results were comparable in the complete case analysis, although the amount of radiographic change was slightly lower for the no baseline hand OA group (Supplementary Figure S1, Supplementary Table S2).

**Table 2.** Amount of radiographic change at 7 years overall, for those free of radiographic osteoarthritis (OA) at baseline, and also separately for baseline hand OA subsets, stratified by gender in the imputed data (n = 706).

	Females			Males		
	n	Adjusted mean*	(95% CI)	n	Adjusted mean*	(95% CI)
Outcome = KL summed score (0–80) †						
Total	438	17.0	(15.8, 18.2)	268	12.5	(11.2, 13.8)
No baseline hand OA	123	9.0	(7.1, 10.9)	98	6.8	(4.7, 8.8)
Thumb base OA	199	21.0	(19.6, 22.5)	104	16.1	(14.0, 18.1)
Nodal IPJ OA	115	26.6	(23.6, 29.5)	37	21.5	(17.7, 25.4)
Generalized hand OA	61	31.6	(28.0, 35.2)	22	27.6	(21.5, 33.7)
Erosive OA	19	40.1	(34.9, 45.3)	3	–	–
Outcome = Number of hand joints with KL grade ≥ 2 (0–20) †						
Total	437	6.7	(6.2, 7.2)	268	5.3	(4.7, 5.8)
No baseline hand OA	123	3.6	(2.9, 4.4)	98	2.8	(2.0, 3.7)
Thumb base OA	198	8.2	(7.7, 8.8)	104	6.6	(5.8, 7.4)
Nodal IPJ OA	114	10.5	(9.3, 11.7)	37	9.4	(7.9, 11.0)
Generalized hand OA	60	12.1	(10.7, 13.4)	22	10.8	(8.9, 12.8)
Erosive OA	18	13.5	(11.6, 15.4)	3	–	–

*Adjusted for baseline value of outcome measure, cohort, age, and time to follow-up; **†**one individual was excluded owing to maximum number of joints affected at baseline (n = 20); – unable to calculate owing to small numbers.

CI, confidence interval; KL, Kellgren–Lawrence; IPJ, interphalangeal joint; No hand OA = KL < 2 in all hand joints; Nodal IPJ OA = KL ≥ 2 in ≥ 2 IPJs (rays 2–5) and ≥ 2 nodes (rays 2–3) across either hand; Thumb base OA = KL ≥ 2 in the first carpometacarpal (1CMCJ) in either hand; Generalized hand OA = KL ≥ 2 in ≥ 1 distal IPJ and ≥ 1 proximal IPJ and ≥ 1 1CMCJ across either hand; Erosive OA = ≥ 2 IPJs (rays 2–5) across either hand.

### Association between metabolic factors and hand OA progression

Overall in all participants, generally non-significant weak associations were found between each of the metabolic factors and the amount of radiographic change for both of the outcomes at 7 years, with adjusted mean differences over 7 years of less than 1 point for the KLsum score or one joint affected with OA (Table 3). These findings were replicated in the complete case analysis (Table 3).

**Table 3.** Association between baseline metabolic factors and hand osteoarthritis (OA) progression at 7 years for all participants and stratified by baseline hand OA subset.

	Analysis based on multiply imputed data				
	All participants (n = 706)	Thumb base OA (n = 303)	Nodal IPJ OA (n = 152)	Generalized OA (n = 83)	Erosive OA (n = 22)
	Outcome = KL summed score (0–80)				
	Adjusted mean difference (95% CI)*				
BMI (kg/m^2^) †	−0.01 (−0.15, 0.13)	−0.11 (−0.33, 0.10)	−0.10 (−0.40, 0.20)	−0.37 (−0.87, 0.13)	−0.47 (−2.13, 1.19)
Hypertension	0.45 (−1.12, 2.03)	0.33 (−2.13, 2.78)	1.82 (−1.59, 5.24)	−0.36 (−6.04, 5.32)	2.49 (−9.26, 14.24)
Diabetes type 2/IFG	0.76 (−1.62, 3.13)	1.50 (−1.75, 4.75)	4.50 (−0.26, 9.25)	3.27 (−2.89, 9.42)	3.05 (−13.56, 19.67)
Dyslipidaemia	0.07 (−1.51, 1.66)	0.72 (−1.61, 3.04)	1.40 (−2.09, 4.89)	1.81 (−3.83, 7.45)	−6.55 (−19.58, 6.47)
No. of metabolic factors (0–4) †	0.02 (−0.57, 0.62)	0.06 (−0.81, 0.93)	0.75 (−0.72, 2.22)	−0.46 (−2.54, 1.62)	−2.09 (−7.94, 3.77)
Metabolic syndrome ‡	0.50 (−1.39, 2.40)	0.87 (−1.90, 3.64)	1.97 (−2.61, 6.54)	−0.81 (−7.85, 6.23)	−0.88 (−17.21, 15.44)
	Outcome = Number of hand joints with KL grade ≥ 2 (0–20) §				
	Adjusted mean difference (95% CI)*				
BMI (kg/m^2^) †	0.01 (−0.05, 0.06)	−0.05 (−0.13, 0.03)	0.01 (−0.11, 0.12)	−0.11 (−0.29, 0.06)	−0.15 (−0.87, 0.57)
Hypertension	−0.01 (−0.63, 0.60)	−0.12 (−1.04, 0.80)	0.34 (−0.92, 1.61)	−0.61 (−2.59, 1.36)	0.34 (−4.58, 5.25)
Diabetes type 2/IFG	0.35 (−0.58, 1.28)	0.67 (−0.58, 1.93)	2.06 (0.25, 3.87)	1.42 (−0.71, 3.56)	2.02 (−6.02, 10.07)
Dyslipidaemia	0.21 (−0.41, 0.83)	0.53 (−0.36, 1.41)	0.67 (−0.60, 1.95)	1.09 (−0.89, 3.08)	−1.02 (−5.65, 3.62)
No. of metabolic factors (0–4) †	−0.01 (−0.25, 0.25)	0.02 (−0.31, 0.35)	0.41 (−0.15, 0.98)	−0.04 (−0.78, 0.70)	−0.50 (−2.76, 1.77)
Metabolic syndrome ‡	0.42 (−0.37, 1.22)	0.68 (−0.38, 1.73)	1.70 (−0.09, 3.50)	0.47 (−1.99, 2.93)	0.25 (−6.05, 6.54)
	Complete case analysis				
	All participants (n = 365)	Thumb base OA (n = 169)	Nodal IPJ OA (n = 85)	Generalized OA (n = 45)	Erosive OA (n = 11)
	Outcome = KL summed score (0–80)				
	Adjusted mean difference (95% CI)*				
BMI (kg/m^2^) †	0.02 (−0.14, 0.19)	−0.17 (−0.45, 0.11)	−0.14 (−0.55, 0.27)	−0.92 (−2.00, 0.15)	–
Hypertension	0.80 (−0.85, 2.44)	0.38 (−2.57, 3.33)	2.15 (−2.65, 6.94)	−4.5 (−15.24, 6.15)	–
Diabetes type 2/IFG	−0.25 (−2.91, 2.41)	0.56 (−3.73, 4.84)	7.78 (1.13, 14.43)	9.46 (−1.98, 20.90)	–
Dyslipidaemia	0.15 (−1.53, 1.83)	1.59 (−1.29, 4.47)	2.34 (−2.56, 7.24)	4.93 (−4.98, 14.85)	–
No. of metabolic factors (0–4) †	0.10 (−0.61, 0.81)	0.08 (−1.02, 1.18)	1.49 (−0.46, 3.44)	0.01 (−3.83, 3.9)	1.12 (−4.59, 6.84)
Metabolic syndrome ‡	0.82 (−1.47, 3.11)	0.82 (−2.75, 4.39)	2.39 (−4.13, 8.92)	−3.60 (−17.06, 9.87)	3.88 (−15.43, 23.18)
	Outcome = Number of hand joints with KL grade ≥ 2 (0–20) § Adjusted mean difference (95% CI)*				
	Complete case analysis				
	All participants (n = 365)	Thumb base OA (n = 169)	Nodal IPJ OA (n = 85)	Generalized OA (n = 45)	Erosive OA (n = 11)
BMI (kg/m^2^) †	0.01 (−0.06, 0.07)	−0.08 (−0.17, 0.02)	−0.01 (−0.15, 0.13)	−0.25 (−0.55, 0.04)	–
Hypertension	0.09 (−0.56, 0.75)	−0.28 (−1.31, 0.76)	0.28 (−1.37, 1.92)	−2.08 (−4.97, 0.81)	–
Diabetes type 2/IFG	−0.24 (−1.30, 0.83)	−0.20 (−1.70, 1.30)	3.35 (1.08, 5.62)	2.94 (−0.13, 6.00)	–
Dyslipidaemia	0.27 (−0.40, 0.94)	1.18 (0.16, 2.19)	1.27 (−0.41, 2.98)	2.44 (−0.33, 5.21)	–
No. of metabolic factors (0–4) †	−0.01 (−0.29, 0.28)	0.02 (−0.38, 0.42)	0.70 (−0.01, 1.39)	0.34 (−0.83, 1.50)	0.04 (−1.54, 1.62)
Metabolic syndrome ‡	0.41 (−0.50, 1.33)	0.39 (−0.90, 1.67)	2.19 (−0.09, 4.48)	−0.29 (−4.36, 3.78)	−1.02 (−5.04, 3.00)

*Estimated from analysis of covariance adjusted for baseline value of outcome measure, cohort, time to follow-up, gender, age, Index of Multiple Deprivation, and smoking status; †per unit increase (all other factors are classed as present/absent); ‡any three of body mass index (BMI) ≥ 30 kg/m^2^, diabetes type 2/impaired fasting glucose (IFG), hypertension, and dyslipidaemia; §one individual was excluded owing to maximum number of joints affected at baseline; – unable to calculate owing to small numbers.

KL, Kellgren–Lawrence; CI, confidence interval; IPJ, interphalangeal joint; Thumb base OA = KL ≥ 2 in the first carpometacarpal (1CMCJ) in either hand; Nodal IPJ OA = KL ≥ 2 in ≥ 2 IPJs (rays 2–5) and ≥ 2 nodes (rays 2–3) across either hand; Generalized hand OA = KL ≥ 2 in ≥ 1 distal IPJ and ≥ 1 proximal IPJ and ≥ 1 1CMCJ across either hand; Erosive OA = ≥ 2 IPJs (rays 2–5) across either hand.

### Association between metabolic factors and progression in hand OA subsets

For the nodal, generalized, and erosive hand OA subsets, adjusted mean differences in the KLsum score at 7 years were consistently higher in individuals with diabetes type 2/IFG than in those without diabetes/IFG in the imputed data, with the adjusted mean differences ranging from 3.05 (−13.56, 19.67) for erosive OA to 4.50 (−0.26, 9.25) for nodal IPJ OA (Table 3). In individuals with nodal OA, the number of affected hand joints at 7 years was also greater in those with diabetes/IFG than in those without diabetes/IFG [adjusted mean difference 2.06 (0.25, 3.87)] (Table 3). Results for erosive OA were similar, although estimates were much less precise owing to the small number in this subset.

The complete case analysis showed similar results but, in addition, dyslipidaemia was positively associated with higher KL summed score and an increase in hand joints affected, with KL ≥ 2 at 7 years in those with thumb base OA, nodal OA, and generalized OA, although this association was statistically significant only for the number of joints affected in thumb base OA (Table 3).

### Association between metabolic factors and incident hand OA

In those free of radiographic OA at baseline, weak non-significant associations were found between the metabolic factors and the amount of radiographic change for the two outcomes at 7 years, adjusted for baseline score and other potential confounders (Table 4). Findings were comparable in the complete case analysis (Table 4).

**Table 4.** Association between baseline metabolic factors and incident hand osteoarthritis (OA) at 7 years in those free of radiographic hand OA at baseline.

	Analysis based on multiply imputed data
	Participants free of hand OA at baseline (n = 221)
	Outcome = KL summed score (0–80)
	Adjusted mean difference* (95% CI)
BMI (kg/m^2^) †	0.05 (−0.18, 0.28)
Hypertension	−0.26 (−2.70, 2.18)
Diabetes type 2/IFG	0.66 (−3.25, 4.57)
Dyslipidaemia	−0.22 (−2.83, 2.40)
No. of metabolic factors (0–4) †	−0.36 (−1.40, 0.68)
Metabolic syndrome ‡	−0.15 (−3.53, 3.22)
	Outcome = Number of hand joints with KL grade ≥ 2 (0–20) §
	Adjusted mean difference* (95% CI)
BMI (kg/m^2^) †	0.02 (−0.08, 0.12)
Hypertension	−0.20 (−1.19, 0.79)
Diabetes type 2/IFG	0.04 (−1.48, 1.55)
Dyslipidaemia	0.16 (−0.92, 1.23)
No. of metabolic factors (0–4) †	−0.15 (−0.59, 0.28)
Metabolic syndrome ‡	−0.07 (−1.52, 1.39)
	Complete case analysis
	Participants free of hand OA at baseline (n = 126)
	Outcome = KL summed score (0–80)
	Adjusted mean difference* (95% CI)
BMI (kg/m^2^) †	0.10 (−0.12, 0.33)
Hypertension	−0.34 (−2.45, 1.78)
Diabetes type 2/IFG	−0.25 (−3.78, 3.29)
Dyslipidaemia	0.04 (−2.35, 2.43)
No. of metabolic factors (0–4) †	−0.16 (−1.13, 0.81)
Metabolic syndrome ‡	−0.15 (−3.26, 2.96)
	Outcome = Number of hand joints with KL grade ≥ 2 (0–20) §
	Adjusted mean difference* (95% CI)
BMI (kg/m^2^) †	0.02 (−0.08, 0.11)
Hypertension	−0.19 (−1.12, 0.74)
Diabetes type 2/IFG	−0.42 (−1.98, 1.13)
Dyslipidaemia	0.08 (−0.98, 1.13)
No. of metabolic factors (0–4) †	−0.16 (−0.59, 0.27)
Metabolic syndrome ‡	−0.11 (−1.47, 1.26)

*Estimated from analysis of covariance adjusted for baseline value of outcome measure, cohort, time to follow-up, gender, age, Index of Multiple Deprivation, and smoking status; †per unit increase (all other factors are classed as present/absent); ‡any three of body mass index (BMI) ≥ 30 kg/m^2^, diabetes type 2/impaired fasting glucose (IFG), hypertension, and dyslipidaemia; §one individual was excluded owing to maximum number of joints affected at baseline.

KL, Kellgren–Lawrence; CI, confidence interval.

## Discussion

Adjusted for baseline values and other covariates, the amount of radiographic change for each outcome at 7 years varied by gender and by baseline hand OA subset, with females and those with nodal, generalized, and erosive OA undergoing greater amounts of progression. Overall, obesity, hypertension, dyslipidaemia, and diabetes type 2/IFG were not found to be associated, either independently or collectively, with the amount of radiographic incidence or progression over 7 years in people with hand symptoms. Trends in the data indicated that the association between metabolic factors and progression may vary by hand OA subset. Diabetes was associated with greater amounts of radiographic progression in those with nodal OA at baseline and possibly implicated in those with generalized and erosive OA.

In this population-based prospective cohort study, descriptive analysis of individuals followed up with hand radiographs at 7 years compared to those lost to follow-up suggests the possibility of attrition bias. As missing data from loss to follow-up could have affected estimates of the associations between metabolic factors and the incidence and progression of radiographic hand OA, MI was undertaken for missing data (37, 46). Therefore, discrepancies between the results of the complete case analysis and the imputed data are likely to be due to selective loss to follow-up, as was noted in the differences in baseline characteristics, and estimates obtained in the MI data were given more credence.

A meta-analysis of cross-sectional studies found an association between the presence of diabetes and OA (6). Hyperglycaemia has been associated with elevated reactive oxygen species and advanced glycation end-products that are thought to lead to low-grade inflammation and oxidative stress, which is believed to damage the chondrocytes (49). We believe that this is the first study to examine diabetes as a risk factor for hand OA progression and find an association in nodal OA. There were also non-significant patterns for increased progression in generalized and erosive OA, although this could be due to the small numbers in the erosive and generalized OA subsets affecting the precision of the estimates. The consistently higher mean differences in the KLsum score at 7 years of between 3 and 5 points in individuals with diabetes compared to those without suggests that diabetes may contribute to progression in specific hand OA subsets, particularly nodal OA. Further examination of the effects of diabetes and the other metabolic risk factors on hand OA progression across different hand OA subsets is required.

While inconsistent findings have been reported in the relation between obesity and hand OA progression (15–17), these early studies were at risk of collider bias due to conditioning on the presence of baseline radiographic hand OA (13, 50, 51). Restricting participants to only those with existing hand OA could lead to biased estimates of the relationship between potential risk factors and hand OA progression. The weak non-significant association between BMI and hand OA progression in the current study is consistent with the results of the Oslo Hand Osteoarthritis Cohort (13). These concordant findings were despite differences in the study population settings (primary vs secondary care), the severity of hand OA at baseline (mean baseline KLsum score 7.8 vs 21.0, respectively), and adjustment for the presence of other metabolic factors in the current study. Risk factors for hand OA could vary for different stages of the disease (52) as associations have been reported between obesity and diabetes, and incident hand OA (10, 18–21), but the current work did not find an association between obesity or diabetes and incident OA over 7 years. This lack of association could be due to the relatively small numbers available in the incident analyses, the small amounts of change that were seen in KLsum score, and the number of joints with KL ≥ 2 over 7 years in this group, and because although individuals were free of radiographic OA, they had hand pain and could have had clinical or pre-radiographic OA.

The lack of association between metabolic factors and the amount of hand OA progression does not necessarily mean that no association exists. The presence of an association is likely to be affected by the time it takes for an exposure to affect the structure of a joint and the amount of exposure that is required to induce change.

This study used a large well-characterized cohort through which selective loss to follow-up was determined and overcome using MI. Attempts were also made to overcome collider bias, which is thought to have been a limitation of previous research (15–17), by including all participants in the analysis, so there was no conditioning for the presence of existing radiographic hand OA, an approach taken by others (13, 53). Associations between metabolic factors and radiographic change were also examined in the subgroup of individuals who were free of OA at baseline, but the findings were unchanged. We, therefore, accept that there is a risk of collider bias if the aim is to estimate the total effect of metabolic factors (the pre-baseline and the baseline status) on disease incidence and progression. However, we feel that our study still makes a useful contribution as our findings highlight that it is unlikely that the change in radiographic OA between baseline and 7 years would be affected by the status of metabolic factors at baseline in a population of middle to later adulthood. Of course, it is still possible that the prevention of these metabolic risk factors would have some effect on radiographic OA change.

There are some limitations that should be acknowledged. Participants were from two studies, but both were general population samples from the same locality, the same data collection was used, and follow-up rates were comparable. It was not possible to differentiate between recently diagnosed and long-standing exposures as we only had consent to access individuals’ medical records for the period 2 years before and after baseline recruitment. Furthermore, objective exposure measurements could not be used as not all individuals had values entered in their medical records. More precise information might reveal differences in risk in those who are more severely affected compared to those individuals just above the threshold for diagnosis. In addition, our analysis did not allow us to differentiate between the presence of a risk factor that is optimally managed and one that is not, which could affect the relation between metabolic factors and hand OA incidence and progression. Finally, while the presence of nodes on rays 2–5 was collected in the CASHA cohort, nodes were only determined on rays 2 and 3 in the CASK cohort to fulfil American College of Rheumatology criteria, which could have led to nodal incidence and progression being underestimated.

## Conclusion

Overall metabolic risk factors were not independently or collectively associated with greater amounts of radiographic hand OA incidence and progression over 7 years. Potential variation was found between the baseline hand OA subsets, with diabetes being a risk factor for radiographic hand OA progression in individuals with nodal, and possibly generalized and erosive OA. Further research is needed to explore differences between hand OA subsets, using objective measures to assess metabolic factors, taking account of the duration of exposures and the extent to which metabolic factors are controlled.

## Supplementary Material

marshall_supplementary_material_110418.pdf

## Supporting information

**Supplementary Table S1**. Variables included in the multiple imputation model.

**Supplementary Figure S1**. Scatter plots showing the relation between baseline and 7 year radiographic scores, stratified by sex in the complete case analysis (n = 388).

**Supplementary Table S2**. The amount of radiographic change at 7 years overall, for those free of radiographic OA at baseline, and also separately for baseline hand OA subsets, stratified by sex in the complete case analysis.
