# Supplementary material for: Metabolic risk factors and the incidence and progression of radiographic hand osteoarthritis: a population-based cohort study
Source: Scand J Rheumatol. 2018 Jun 28;48(1):52–63. doi: 10.1080/03009742.2018.1459831 (PMC6319183; doi:10.1080/03009742.2018.1459831)
Supplement: marshall_supplementary_material_110418.pdf [file IRHE_A_1459831_SM8242.pdf]

Supplementary table S1. Variables included in the multiple imputation model

Concept	Measurement	Baseline	7-years	Included in subsequent analysis
Descriptive & sociodemographic	Sex (Female, Males)	•		•
	Age (years)	•		•
	Cohort (CASHA, CASK)	•		•
	Follow-up time (months)		•	•
	Smoking status (Never, Ex, Current)	I = 0.3%		•
	Index of Multiple Deprivation	I = 0.3%		•
	Further education (Yes, No)	•		
	Socioeconomic status[Office for National Statistics 2000]	•		
Metabolic factors (2 years before and after baseline)	BMI	I = 0.3%		•
	Hypertension (Present, Absent)	I = 6.5%		•
	Diabetes type 2 or impaired fasting glucose (Present, Absent)	I = 6.5%		•
	Dyslipidaemia (Present, Absent)	I = 6.5%		•
	No. metabolic factors (0-4)	I = 6.5%		•
	Metabolic Syndrome (Present, Absent) †	I = 6.5%		•
	No. days statins prescribed	I = 6.5%		
Radiographic OA	KLsum score (0-80)	•	I = 45.0%	•
	No. joints with OA (KL≥2) (0-20)	•	I = 45.0%	•
Hand characteristics and symptoms	Hand problem in the past 12 months (Yes, No)	•	•	
	Hand pain in the past 12 months (Yes, No)	•	•	
	Side of pain in past 12 months (Right, Left, Both)	•	•	
	Duration of hand problem (months)	•		
	Duration of pain in past 12 months (<7 days, 1-4 weeks, 1-3 months, >3 months)	•	•	
	Frequency of pain in past 1 month (no, few, some, most, all days)	•	•	
	AUSCAN pain, function & stiffness subscales[Bellamy 2002] (0-20; 0-36; 0-4 respectively)	•	•	
	Thumb base OA (Present, Absent)	•	•	•
	Nodal OA (Present, Absent)	•	•	•
	Generalised OA (Present, Absent)	•	•	•
	Erosive OA (Present, Absent)	•	•	•
General, physical and mental health	SF12 physical and mental component scores[Ware 1996] (0-100 each)	•		
	SF36 physical functioning scale[Ware & Sherborne 1992] (0-100)	•		
	HADS anxiety and depression subscales[Zigmond & Snaith 1983](0-21 each)	•		
Self-reported comorbidities and health problems	Raised blood pressure (Present, Absent)	•		
	Diabetes (Present, Absent)	•		
	Chest problems (Present, Absent)	•		
	Heart problems (Present, Absent)	•		
	Deafness (Present, Absent)	•		
	Problems with eyesight (Present, Absent)	•		
	A fall or falls (Present, Absent)	•		
	Difficulty remembering things (Present, Absent)	•		
	Cough with spit (Present, Absent)	•		
	Breathless when walking (Present, Absent)	•		
	Dizziness or unsteadiness (Present, Absent)	•		
	Weakness in an arm or leg (Present, Absent)	•		

BMI, Body Mass Index; KL, Kellgren Lawrence; AUSCAN, Australian-Canadian Hand Osteoarthritis Index; SF12, Short Form 12; HADS, Hospital Anxiety and Depression Scale; SF36, Short Form 36; I, Data that was imputed and the proportion. † Metabolic Syndrome = Any three of BMI≥30kg/m^2, diabetes type 2/IFG, hypertension, dyslipidaemia. Thumb base OA = KL≥2 in the 1CMCJ in either hand; Nodal IPJ OA = KL≥2 in ≥2 IPJs (rays 2-5) & ≥2 nodes (rays 2-3) across either hand; Generalised hand OA = KL≥2 in ≥1 distal IPJ & ≥1 proximal IPJ & ≥1 1CMCJ across either hand; Erosive OA = E or R phase in ≥2 IPJ (rays 2-5) across either hand.

References: Bellamy N, et al. Dimensionality and clinical importance of pain and disability in hand osteoarthritis: Development of the Australian/Canadian (AUSCAN) Osteoarthritis Hand Index. Osteoarthritis Cartilage 2002;10:855-62. Office for National Statistics (ONS). The National Statistics Socioeconomic Classification User Manual (version 1). London: Office for National Statistics. 2002. Ware J Jr, et al. A 12-Item Short-Form Health Survey: construction of scales and preliminary tests of reliability and validity. Med Care 1996;34:220-33. Ware JE Jr, Sherbourne CD. The MOS 36-item Short-Form health survey (SF-36). I. Conceptual framework and item selection. Med Care. 1992;30:473-83. Zigmond AS, Snaith RP. The hospital anxiety and depression scale. Acta Psychiatr Scand 1983;67:361-70.

Supplementary figure S1. Scatter plots showing the relation between baseline and 7-year radiographic scores, stratified by sex in the complete case analysis (n = 388)

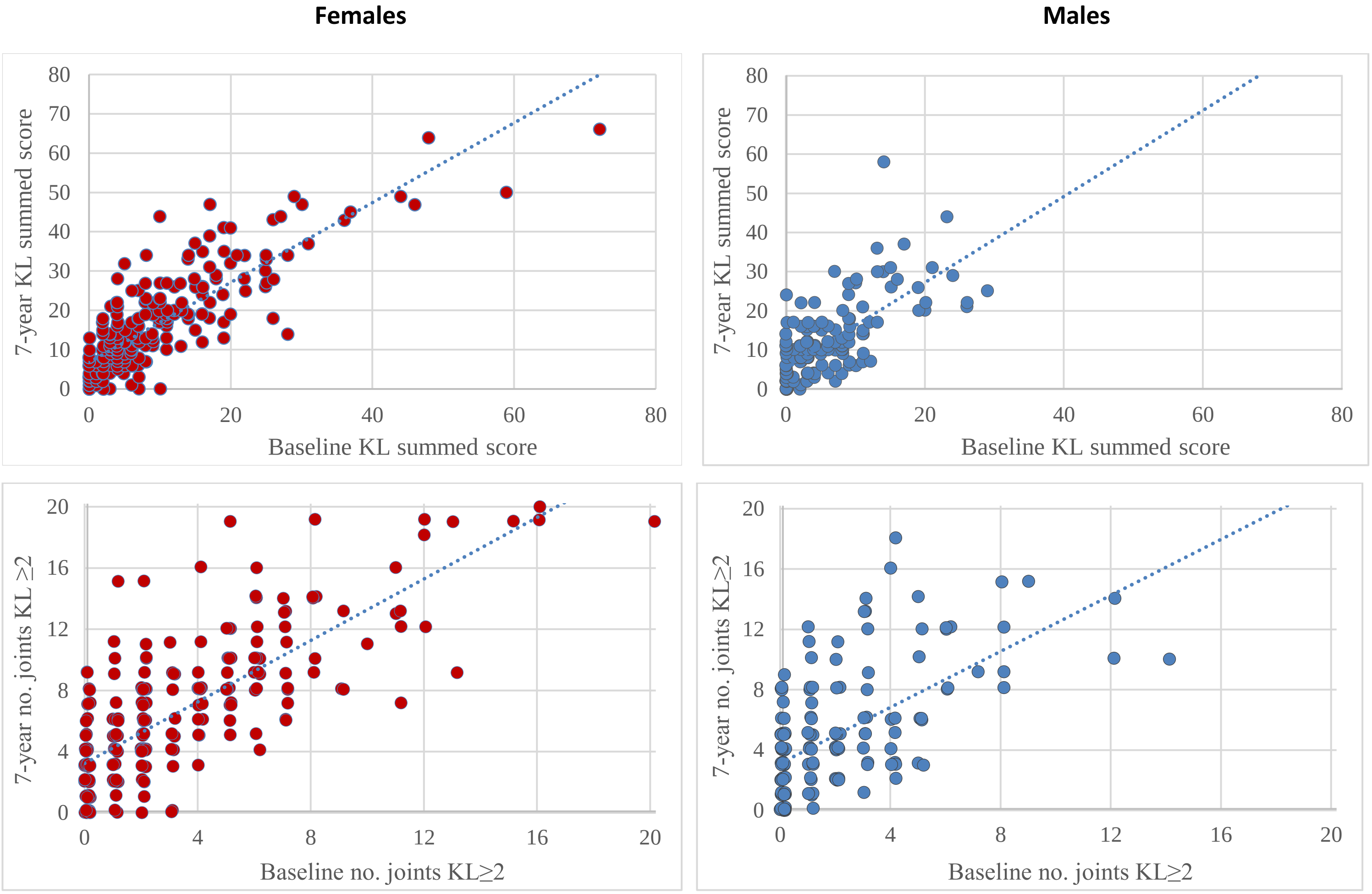

KL, Kellgren Lawrence. Jittering has been used to allow better visualisation of overlapping markers.

Supplementary table S2. The amount of radiographic change at 7-years overall, for those free of radiographic OA at baseline, and also separately for baseline hand OA subsets, stratified by sex in the complete case analysis

	Females			Males		
	**Outcome = Kellgren-Lawrence summed score (0-80)**					
	n	Adjusted mean*	(95%CI)	n	Adjusted mean*	(95%CI)
Total	233	16.5	(15.5, 17.5)	155	11.1	(10.0, 12.1)
No baseline hand OA	62	5.6	(4.1, 7.2)	64	4.0	(2.5, 5.6)
Thumb base OA	115	21.3	(19.9, 22.8)	54	16.1	(13.4, 18.9)
Nodal IPJ OA	62	27.9	(24.2, 31.6)	23	20.7	(15.9, 25.5)
Generalised hand OA	32	33.0	(28.5, 37.5)	13	27.2	(14.9, 39.4)
Erosive OA	10	42.8	(38.3, 47.3)	1	-	-
	**Outcome = Number of hand joints with Kellgren-Lawrence Grade≥2 (0-20) †**					
	n	Adjusted mean*	(95%CI)	n	Adjusted mean*	(95%CI)
Total	232	6.5	(6.1, 6.9)	155	4.8	(4.3, 5.2)
No baseline hand OA	62	2.4	(1.7, 3.0)	64	1.7	(1.0, 2.5)
Thumb base OA	114	8.4	(7.8, 8.9)	52	6.8	(5.8, 7.7)
Nodal IPJ OA	61	11.1	(9.9, 12.3)	23	9.2	(7.3, 11.1)
Generalised hand OA	31	12.7	(11.2, 14.1)	13	11.0	(7.7, 14.4)
Erosive OA	9	13.3	(11.5, 15.0)	1	-	-

*95% CI, 95% Confidence interval; * adjusted for baseline value of outcome measure, cohort, age, time to follow-up; † One individual excluded due to maximum number of joints affected at baseline (n=20). No hand OA = KL<2 in all hand joints; Nodal IPJ OA = KL≥2 in ≥2 IPJs (rays 2-5) & ≥2 nodes (rays 2-3) across either hand; Thumb base OA = KL≥2 in the 1CMCJ in either hand; Generalised hand OA = KL≥2 in ≥1 distal IPJ & ≥1 proximal IPJ & ≥1 1CMCJ across either hand; Erosive OA = ≥2 IPJ (rays 2-5) across either hand.*
